# Development and Evaluation of Novel Metformin Derivative Metformin Threonate for Brain Ischemia Treatment

**DOI:** 10.3389/fphar.2022.879690

**Published:** 2022-06-21

**Authors:** Gufang Zhang, Shuangshuang Chen, Jia Jia, Chun Liu, Weipeng Wang, Hongjian Zhang, Xuechu Zhen

**Affiliations:** ^1^ Jiangsu Key Laboratory of Neuropsychiatric Diseases, Department of Pharmacology, College of Pharmaceutical Sciences, Soochow University, Suzhou, China; ^2^ Department of Pharmaceutical Analysis, College of Pharmaceutical Sciences, Soochow University, Suzhou, China

**Keywords:** ischemic stroke, metformin threonate, microglia, neuroinflammation, functional recovery

## Abstract

Epidemiologic data reveal that diabetes patients taking metformin exhibit lower incidence of stroke and better functional outcomes during post-stroke neurologic recovery. We previously demonstrated that chronic post-ischemic administration of metformin improved functional recovery in experimental cerebral ischemia. However, few beneficial effects of metformin on the acute phase of cerebral ischemia were reported either in experimental animals or in stroke patients, which limits the application of metformin in stroke. We hypothesized that slow cellular uptake of metformin hydrochloride may contribute to the lack of efficacy in acute stroke. We recently developed and patented a novel metformin derivative, metformin threonate (SHY-01). Pharmacokinetic profile *in vivo* and in cultured cells revealed that metformin is more rapidly uptaken and accumulated from SHY-01 than metformin hydrochloride. Accordingly, SHY-01 treatment exhibited more potent and rapid activation of AMP-activated protein kinase (AMPK). Furthermore, SHY-01 elicited a stronger inhibition of microglia activation and more potent neuroprotection when compared to metformin hydrochloride. SHY-01 administration also had superior beneficial effects on neurologic functional recovery in experimental stroke and offered strong protection against acute cerebral ischemia with reduced infarct volume and mortality, as well as the improved sensorimotor and cognitive functions in rats. Collectively, these results indicated that SHY-01 had an improved pharmacokinetic and pharmacological profile and produced more potent protective effects on acute stroke and long-term neurological damage. We propose that SHY-01 is a very promising therapeutic candidate for cerebral ischemic stroke.

## Introduction

Stroke is one of the major causes of death and adult disability worldwide. It is generally believed that post-stroke inflammation-induced secondary injury or infection remains to be an essential challenge that influences the recovery in stroke patients (Kluge et al., 2019; Phipps and Cronin, 2020; Powers, 2020). In addition to neuroprotection and neurogenesis, targeting microglia to modulate inflammation is one of the promising therapeutic approaches for stroke treatment.

AMP-activated protein kinase (AMPK) is a conserved serine/threonine kinase that serves as a critical sensor in the regulation of cellular energy homeostasis and metabolic pathways. AMPK activation inhibits neuroinflammation and promotes microglia/macrophage polarization toward the M2 phenotype, a resolving-type microglia/macrophage that promotes tissue recovery through producing anti-inflammatory cytokines and clearing apoptotic cells or debris. In contrast, M1 phenotype represents pro-inflammatory microglia/macrophages that exacerbate tissue injury by releasing massive inflammatory cytokines during aseptic inflammation (Jiang et al., 2018; Wang et al., 2018; Steinberg and Carling, 2019). Metformin is an established AMPK activator and is widely used as the first-line drug for type 2 diabetes treatment. It is interesting to note that epidemiological studies have shown that diabetic patients undergoing long-term metformin treatment had a significantly lower risk of stroke incidence or improved functional recovery that was independent of the glucose-lowering effect of the drug (Ashabi et al., 2015; Castilla-Guerra et al., 2018; Westphal et al., 2020). The beneficial effects of metformin were also observed in stroke patients without diabetes (Jadhav et al., 2006). Consequently, a potential therapeutic application of metformin in stroke has been proposed (Nantsupawat et al., 2020). In support of this proposal, we previously provided the first experimental evidence that activation of AMPK by post-stroke chronic metformin administration improves the long-term neurologic functional recovery in experimental stroke mice induced by transient middle cerebral artery occlusion (tMCAO) (Jin et al., 2014). This finding was confirmed by other studies (Leech et al., 2019; Zemgulyte et al., 2021). We further found that metformin could regulate microglia-mediated inflammation through metabolic reprogramming. In addition, metformin was recently shown to modulate autophagy and ultimately contribute to neuronal survival (Yan et al., 2017).

However, it is interesting to note that although post-stroke chronic metformin administration improved the long-term neurologic functional recovery, metformin treatment failed to elicit significant beneficial effects on acute ischemic stroke either in experimental studies or in clinical practice (Mima et al., 2016; Zhao et al., 2019). The lack of benefits in acute ischemia limits the application of metformin.

It is well established that metformin is unable to cross the cell membrane via passive diffusion due to its hydrophilicity (logD −6.13 at pH 7.0) and its positively charged state under physiologic conditions (pKa 12.4), and that it requires cation transporters to enter the cells (Dewhirst and Fry, 2018; Lu et al., 2019). Consequently, it takes hours for metformin (metformin hydrochloride) to reach the effective intracellular concentration needed to activate AMPK (Lu et al., 2019; Van Nostrand et al., 2020). We thus hypothesize that the lack of protective effect of metformin hydrochloride on acute ischemic stroke may be attributed to the slow accumulation of intracellular metformin, which delays pharmacological response, beyond the effective therapeutic window. We thus thought to optimize the structure or acid radical of the currently used metformin. L-Threonate, (2R,3S)-2,3,4-Trihydroxybutanoate, is a natural sugar-acid that is found in plasma and the aqueous humor of the eye (Dewhirst and Fry, 2018). It was shown that, threonate promotes Mg^2+^ influx to cells, moreover; threonate has the ability to enhance its conjugated compound intracellular bioavailability (Kim et al., 2020; Slutsky et al., 2010). We have designed and synthesized several groups of hybrids of metformin and found that metformin threonate (SHY-01) exhibited more rapid and potent activation of AMPK than the traditionally used hydrochloride acid of metformin (Zhen et al., 2019). Our results indicate that, compared to metformin hydrochloride, metformin threonate not only promotes improved functional recovery when chronically administrated to experimental ischemic stroke rats but also produced protection on acute ischemic stroke by decreasing the mortality and infarct volume. The superior beneficial effects of SHY-01 make it a promising candidate for stroke treatment.

## Materials and Methods

### Animals and Transient Middle Cerebral Artery Occlusion Surgery

All animal experimental procedures were approved by the animal welfare committee of Soochow University and followed the guidance of the NIH for the Care and Use of Laboratory Animals. Male Sprague-Dawley rats, weighing 250–280 g, were purchased from SLAC Laboratory Animals (Shanghai, China). For the model of transient MCAO (tMCAO), rats were anesthetized with isoflurane (3% for induction and 2% for surgical procedure) in a mixture of oxygen/nitrous oxide (30%/70%). Focal cerebral ischemia was produced by 1.5 h of middle cerebral artery occlusion (MCAO) followed by reperfusion via an intraluminal suture technique as previously reported (Jin et al., 2014). Sham-operated rats underwent the same surgery except for the suture insertion. After surgery, the body temperature of rats was maintained at 37°C ± 0.5°C with a homoeothermic blanket control unit (Beijing Clinontech Co., LTD., China). The rats that exhibited neurologic deficits, including unbalanced walking and spastic limb tone, were selected for the subsequent studies.

For the observation of the treatment effect, rats were intraperitoneally (*i.p*.) injected with 0.1 ml volume of SHY-01 (5, 15, and 50 mg/kg) or metformin (50 mg/kg) dissolved in saline every 24 h post-reperfusion until sacrifice.

### Experimental Design

To analyze the effective dosage of SHY-01 on ischemic rats, a total number of 90 animals were subdivided randomly into six groups (each comprising 15 animals): sham group, rats subjected to tMCAO treated with the vehicle group, rats subjected to tMCAO treated with 5, 15, and 50 mg/kg SHY-01 (*i.p.*), respectively, rats subjected to tMCAO treated with 50 mg/kg metformin hydrochloride (*i.p.*). SHY-01 or metformin hydrochloride was intraperitoneally injected into rats every 24 h post-reperfusion for seven constitutive days. Survival was recorded daily.

To evaluate the time window of SHY-01 treatment during cerebral ischemia, rats were divided into five groups: sham rats (*n* = 7 rats), rats subjected to tMCAO treated with vehicle (*n* = 8 rats), rats subjected to tMCAO treated with 50 mg/kg SHY-01 (*i.p.*) at 2, 6, or 24 h post-reperfusion (*n* = 8 rats/group). The neurologic score was measured and brains were assessed 3 days after reperfusion.

To compare the effect of SHY-01 and metformin on the ischemic stroke, rats were divided into four groups (each comprising eight animals): sham group, rats subjected to tMCAO treated with vehicle, rats subjected to tMCAO treated with 50 mg/kg SHY-01 (*i.p.*), or metformin every 24 h. The neurologic score was measured and brains were assessed 3 days after reperfusion. In the long-term recovery of ischemic rats treated with SHY-01 or metformin, there were 12 rats per group at the endpoint (28 days).

### Behavioral Test

Modified neurological severity scores (mNSS) were based on sensory, motor, balance, and reflex tests and graded on a scale of 0–18 (normal score 0, maximal deficit score 18) as previously described (Jin et al., 2014), mNSS test was assessed blindly at 3, 7, 14, and 28 days after ischemic surgery to evaluate neurological deficits. Morris test was evaluated for learning and memory deficits for long-term function recovery determination. For this evaluation, rats were trained for 2 days and then the basal behavioral tests were administered 1 day before tMCAO, tests were continually performed at 3, 7, 14, and 28 days after tMCAO.

The Morris water maze is a large round tub (160 cm diameter, 50 cm height) of opaque water (made white with powdered milk) with small hidden platforms located 1–2 cm under the water surface. The pool was divided into four quadrants and the platform was placed in one of the quadrants. After acclimating to the environment for 30 min, the rat was placed into the pool facing the side wall at one of four random start locations in each quadrant. Each rat was allowed to swim to the hidden platform and the time period of the rat found on the platform was recorded to evaluate learning and memory skills. If the rat could not find the platform within 60 s, it was allowed to be guided to the platform and remained on it for 10 s. Rats were trained for 3 days before tMCAO. The latency time of the average of the four trails to reach the platform was recorded.

A rope climbing test was used to test the rats’ forelimbs and hindlimbs coordination. A rope of 1.5 cm in diameter was dropped vertically from a platform (15-cm length, 50-cm width). The rats were trained for 2 days before tMCAO surgery. The score criterion was defined as follows: “0”—rat can climb to the platform within 10 s without any stimulation; “1”—rat can climb to the platform within 15 s without any stimulation; “2”—rat can climb to the platform within 30 s with less than five times stimulation; “3”—rat can climb to the platform within 60 s with less than five times stimulation; “4”—rat can climb to the platform more than 60 s with less than five times stimulation or climb to the platform within 60 s with more than five times stimulation; and “5”—rat is unable to climb to the platform even with repeated stimulation.

### Antibodies and Reagents

Metformin threonate (SHY-01) was synthetized according to the application patent of “Application of metformin salt in cerebral ischemic stroke” (application number: CN110037998A); Compound C, TTC, MTT, and LPS (*E. Coli* 0111: B4) was purchased from Sigma-Aldrich (St. Louis, United States). DMEM, DMEM F12, DNase I, and trypsin were purchased from Invitrogen (Camarillo, United States). FBS was purchased from Gibco (Carlsbad, United States), anti-total AMPK and phosphorylated AMPK rabbit antibodies were purchased from Cell Signaling Technology (Danvers, United States), IRDye^®^ 800CW Donkey anti-Rabbit IgG (H + L), and IRDye^®^ 680RD Goat anti-Mouse IgG (H + L) were purchased from LI-COR, Inc. (Nebraska, United States), anti-Iba1 polyclonal rabbit antibody was purchased from Wako (Osaka, Japan), goat anti-rabbit IgG with Alexa Fluor 488, DAPI, and TRIzol™ Reagent were purchased from Invitrogen (Carlsbad, United States). PrimeScript™ RT reagent Kit was purchased from Takara Biomedical Technology (Dalian, China).

### Histological Analysis of Rat Brain

Rats were sacrificed at 3 or 28 days after tMCAO, brains were collected and dissected on ice, sectioned into 2 mm coronal sections and the sections were incubated with 2% TTC (2,3,5-triphenyl tetrazolium chloride) phosphate buffer for 20 min at 37°C in the dark. Normal brain tissue exhibited red, whereas the infarct area exhibited white. The percentage of the infarct areas to the total brain areas was calculated by morphometric analysis with Image-Pro Plus. Residual brain tissue of rats subjected to tMCAO at 28 days after reperfusion was used to evaluate the long-term recovery effect of SHY-01 (Zhu et al., 2021). Brain atrophy (%) = (Brain volume of contralateral−Residue brain volume of ipsilateral)/Brain volume of contralateral × 100%.

### Immunofluorescence

Rats were anesthetized and brains were dissected after perfusion with PBS, then brains were extracted and kept in 4% paraformaldehyde overnight followed by 30% sucrose for cryoprotection. 20-μm thick brain sections were blocked by 5% normal donkey serum and then incubated with anti-Iba1 polyclonal rabbit antibody (1:500) overnight at 4°C. After three washes with PBST (0.1% Tween 20 in PBS), sections were incubated with Alexa 488-conjugated donkey anti-rabbit (Invitrogen) antibody, Hoechst 33258 (Invitrogen) was used to stain nuclei. Images were obtained by confocal microscopy (LSM710, Carl Zeiss, and Germany).

For the detection of apoptotic cells, *In Situ* Cell Death Detection Kit (12156792910, Roche) was used. Brain sections were fixed in 4% paraformaldehyde and permeabilized with 0.1% Triton X-100 on ice, then incubated with TUNEL for 60 min at 37°C in the dark. Sections were washed three times and incubated with PI for 5 min at room temperature. Apoptotic cells were present in red. The percentage of TUNEL-positive cells among total cells (Hoechst positive cells) per field in five sections from each rat was counted, and the mean number of TUNEL cells in the field of view was calculated for each rat. Images were obtained by confocal microscopy (Carl Zeiss, LSM710, and Germany).

### Real-Time PCR

Total RNA was extracted from microglia or brain cortex using Trizol reagent (Invitrogen). Isolated RNA was reverse transcribed into cDNA using cDNA synthesis kit (Takara) following standard protocols. Real-time quantitative PCR (qPCR) was performed using synthetic primers and SYBR Green (Invitrogen) on an Applied Biosystems 7500 Fast Real-Time PCR System (ThermoFisher). The primer sequences for each gene are listed as follows: rat *tnf*-α (Forward: 5′- TTC​CCA​AAT​GGG​CTC​CCT​CT; Reverse: 5′- GTG​GGC​TAC​GGG​CTT​GTC​AC), rat *il*-1β (Forward: 5′- TCC​AGG​ATG​AGG​ACC​CAA​GC; Reverse: 5′- TCG​TCA​TCA​TCC​CAC​GAG​TCA), rat *inos* (Forward: 5′- AGG​CCA​CCT​CGG​ATA​TCT​CT; Reverse: 5′- GCT​TGT​CTC​TGG​GTC​CTC​TG), rat *gapdh* (Forward: 5′- GGT​TCC​GGT​TTG​TGG​AGC​AG; Reverse: 5′- TCC​GTT​TGC​ATT​GCC​CAG​TA). Gene expression level was calculated relative to *gapdh* expression level.

### Cell Culture

BV2 cells, the immortalized microglia-like cell lines, were cultured in Dulbecco’s modified Eagle’s medium (DMEM, Invitrogen), supplemented with 10% fetal bovine serum (FBS, Gibco), 100 U/ml penicillin and 100 μg/ml streptomycin (Invitrogen) at 37°C in an atmosphere of 95% air and 5% CO_2_. When reached 80% confluence, cells were seeded in 12-well or 6-well plates to perform further experiments.

Primary rat cortical microglia cells were prepared from newborn to 2–3 days-old SD strains. After removing the meningeal of rats, the entire cortices were dissected and placed into 0.25% trypsin (Invitrogen) and DNase I (0.1 mg/ml, Sigma-Aldrich) at 37°C, DMEM/F12 (Invitrogen) supplemented with 10% FBS was added to trypsin 15 min later to stop the digestion. The supernatant was discarded after centrifuging at 1,100 × rpm for 10 min, the pellet was resuspended, and cortices were fully dissociated by pipetting, the cell suspension was filtered through a 70 μm mesh and transferred into a 75-cm^2^ culture flask with poly-L-lysine (PLL, Sigma-Aldrich) coated. Flasks were incubated at 37°C and 5% CO_2_, and half of the culture media were changed every 2 days, after 2 weeks of culture, primary microglia were harvested by shaking the flask for 2 h at 200 × rpm, and then seeded into new plates pre-coated with PLL.

Primary rat cortical neurons were obtained from E16 to E18 embryos, and the step of brain tissue digestion was similar as primary cortical microglia harvested (Chen L. et al., 2020). The culture medium was replenished with Neurobasal medium (Life Technologies) supplemented with 2% B27 (Life Technologies) 5 h after isolation, and half of the culture media was changed every 2 days, after 6 days of culture, and the neurons were used for further experiments.

### Oxygen and Glucose Deprivation

Primary rat cortical neurons were pre-treated with a series of concentrations of SHY-01 or metformin for 24 h, then the complete medium was replaced with glucose-free Earle’s balanced salt solution (EBSS, pH 7.4). Cells were transferred to a hypoxia incubator with 1% oxygen and 5% CO_2_ for 3 h. After OGD, the medium was replaced, and cells were incubated for an additional 24 h under normal conditions with 95% air and 5% CO_2_ at 37°C. Control cells were not submitted to OGD and were maintained under normal conditions. Cell viability was evaluated by the MTT assay described previously (Han et al., 2020).

### Enzyme-Linked Immunosorbent Assay and Nitrite Measurement

Supernatants of BV-2 microglia cells were analyzed by enzyme-linked immunosorbent assay (ELISA) for TNF-α (ThermoFisher, 88-7324-22) and IL-1β (ThermoFisher, 88-7013-22). BV-2 cells were pre-incubated with SHY-01 or metformin hydrochloride for 30 min followed by LPS stimulation, supernatants were collected after 24 h and centrifuged at 500 × g for 10 min to get rid of cells. Protein levels of TNF-α and IL-1β in cell supernatants were quantified as previously described (Han et al., 2019). The level of NO was quantified with Griess reagent (1% sulfanilamide, 0.1% naphthyl ethylene, 2% phosphoric acid) described before (Jin et al., 2014). In brief, 50 μl cell supernatants were mixed with 50 μl Griess regent in 96-well-plate and reacted for about 5–10 min at room temperature. Read plate at 540 nm by a Microplate reader (Infinite M200, Molecular Device). 0–150 μM sodium nitrite was used as a standard to prepare a standard cure.

### Measurement of Lactate Dehydrogenase Activity

Primary rat microglia were pre-treated with 1 mM SHY-01 or metformin hydrochloride for 3 h followed with or without LPS (100 ng/ml) treatment for 24 h, supernatants were then collected as a conditional medium and added to a primary rat cortical neuron culture. The neuron culture medium was collected and LDH was measured by the LDH assay kit (BioAssay Systems) according to the manufacturer’s instructions.

### Cellular Pharmacokinetics of SHY-01 in Microglia

BV-2 cells were seeded on a 24-well plate and cultured under normal conditions with 95% air and 5% CO_2_ at 37°C. After washing with HBSS three times, cells were incubated with HBSS for 0.5 h and then replaced with a series of dose of SHY-01 or metformin hydrochloride dissolved in HBSS. At indicated time points, cells lysis were achieved by the addition of ultrapure water and placed at −80°C for repeated freeze-thaw cycles. Quantification of dissociating metformin at its endogenous level was performed by high-performance liquid chromatography-tandem mass spectrometry (LC-MS/MS)-based bioanalytical method in the positive-ion mode (ESI) using API4000 Qtrap Mass Spectrograph (SCIEX, USA) and with separation conducted on Agela Venusil XBP- C18 column (2.1*50 mm, 5 μm particle size, Tianjin, China).

### Pharmacokinetics and Brain Distribution

Forty-two male rats were intraperitoneally injected with SHY-01 or metformin hydrochloride at 50 mg/kg. After 0.5, 1, 2, 4, 8, 12, or 24 h, blood samples were collected in tubes with EDTA as anticoagulant regent and were centrifuged at 6,000 × g for 5 min. Plasma samples were then transferred to new tubes and stored at −80°C until analysis. Brain tissues were harvested at 1 or 2 h after administration and stored at −80°C freezer immediately. The concentrations of metformin in plasma and brain tissue were determined using high-performance liquid chromatography-tandem mass spectrometry (LC-MS/MS). Briefly, 25 μl plasma was added to 100 μl acetonitrile containing internal standard, and the sample was vortexed for 2 min and then centrifuged at 13,000 × g for 10 min. Subsequently, a 10 μl volume of the supernatant was used for the determination of metformin concentration by LC-MS/MS. Brain samples were weighted and homogenized in the triple volume of saline, followed by the ultrasonication for 15 min 100 μl of acetonitrile containing internal standard was added to 25 μl of the sample, which was vigorously vortexed for 2 min and then centrifuged at 13,000 × g for 10 min. After that, 10 μl of the supernatant was used for the determination of metformin concentration by LC-MS/MS.

### Statistical Analysis

Data are presented as the means ± the standard deviation (SD) with the exception of the neurologic scores and the Morris test data, which are expressed as the medians ± SEM. Statistical analyses were conducted by GraphPad Prism 8.0 (GraphPad Software, Inc.). Comparisons between two groups were performed using Two-tailed Unpaired Student’s *t*-tests and one-way ANOVAs with Dunnett’s post hoc tests were used for multiple groups comparison. Survival analysis was performed with Kaplan-Meier analysis. *p* < 0.05 was considered statistically significant.

## Results

### Metformin Threonate (SHY-01) Rapidly Activates AMP-Activated Protein Kinase in Microglia

A series of novel derivatives, metformin threonate, metformin tartrate, metformin citrate, metformin mesylate, metformin maleate, and metformin hydrobromide were synthesized. After initial extensive screening for cell toxicity and AMPK activation, we found that SHY-01 (metformin threonate) exhibited the strongest potency on AMPK activation with a better safety profile (Zhen et al., 2019).

**FIGURE 1:**
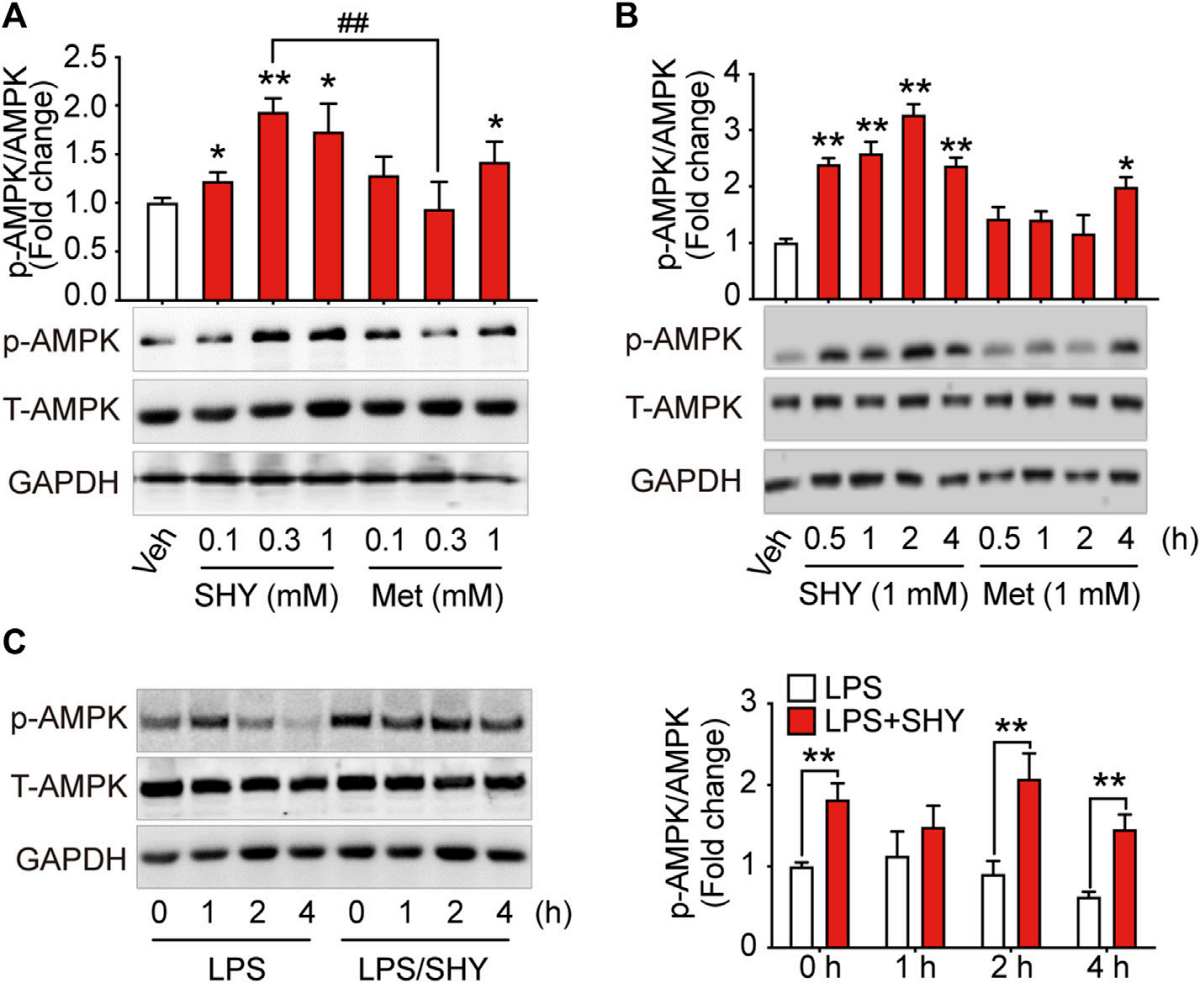
SHY-01 rapidly activates AMPK in microglia. **(A)** BV-2 microglial cells were treated with various concentrations of SHY-01 (SHY) or metformin hydrochloride (Met) for 1 h, PBS was used as vehicle (veh) control. Phosphorylation of AMPK (Thr 172) was detected by Immunoblot. ∗*p* < 0.05, ∗∗*p* < 0.01 versus the vehicle control, ^##^ *p* < 0.01 represent 0.3 mM Met versus 0.3 mM SHY-01. **(B)** BV-2 microglial cells were treated with 1 mM SHY-01 (SHY) or Met for indicated time points. ∗*p* < 0.05, ∗∗*p* < 0.01 versus the vehicle control. In **(A,B)**, phosphorylated (p)-AMPK (Thr 172) protein levels were expressed relative to vehicle (veh) control as mean ± SD (*n* = 3) and data were analyzed using one-way ANOVA followed by Dunnett post hoc tests. **(C)** BV-2 microglial cells were pre-incubated with 1 mM SHY-01 (SHY) followed with LPS (200 ng/ml) challenge for 0, 1, 2, or 4 h. Cells were collected for Immunoblot analysis of p-AMPK (Thr 172), total AMPK and GAPDH. Relative p-AMPK protein levels were expressed as mean ± SD (*n* = 3), data were analyzed using two-way ANOVA followed by Dunnett’s post hoc tests. ∗∗*p* < 0.01 versus the LPS group.

As shown in Figure 1, when BV-2 microglial cells were incubated with different concentrations of SHY-01 or metformin hydrochloride. (Metformin) for 1 h, activation of AMPK as detected by p-AMPK was already observed in SHY-01-treated microglia at 0.3 and 1.0 mM, while the effective dose of metformin hydrochloride was up to 1.0 mM. Moreover, p-AMPK level in microglia was significantly higher with 0.3 mM SHY-01 incubation compared to the same concentration of metformin hydrochloride (Figure 1A). Importantly, the time-course study revealed that the onset time of AMPK activation by 1 mM SHY-01 was 0.5 h, whereas the earliest onset time for metformin treatment was 4 h (Figure 1B). Furthermore, SHY-01 treatment could significantly reverse the LPS-reduced AMPK phosphorylation on microglia (Figure 1C). These data revealed that, although both SHY-01 and metformin activated AMPK, SHY-01 produced a faster and more potent activation.

### SHY-01 Elicits a More Potent Inhibition on Neuroinflammation *via* AMP-Activated Protein Kinase Activation

**FIGURE 2:**
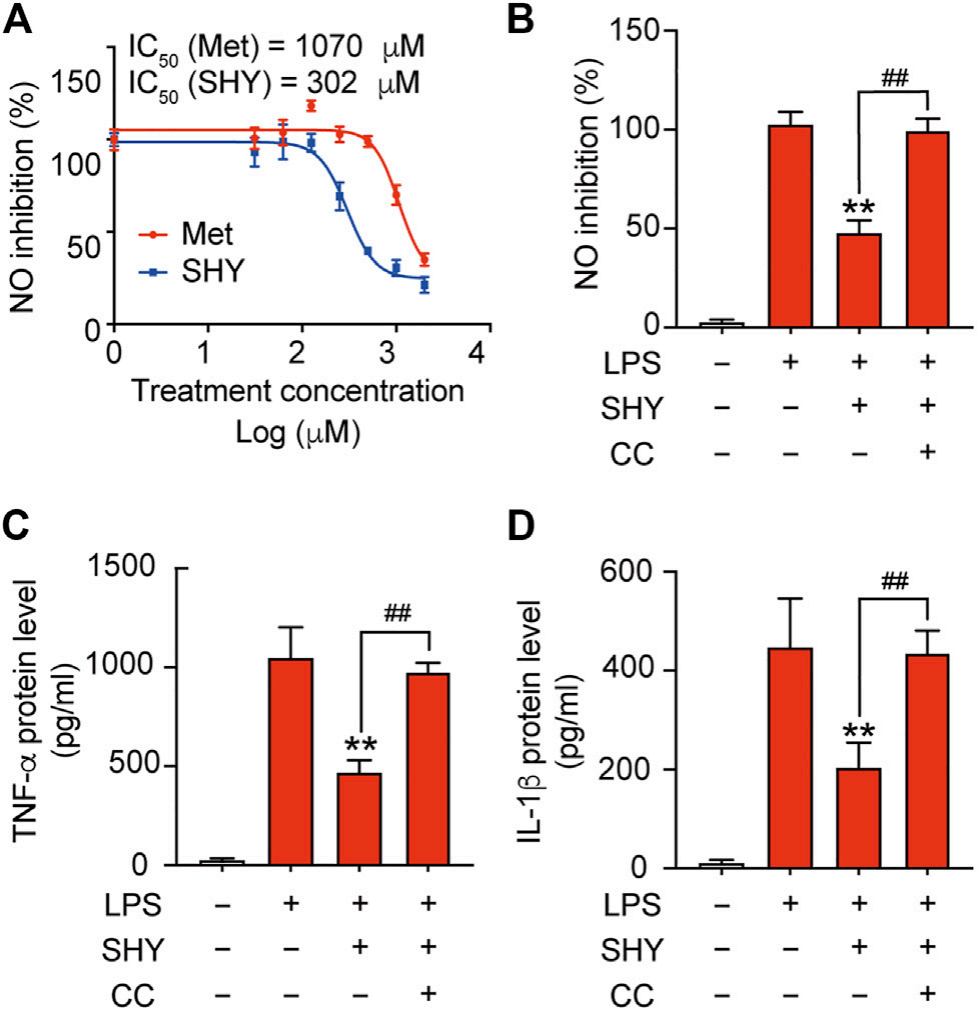
SHY-01 inhibits LPS-induced inflammation through AMPK activation. **(A)** BV-2 microglial cells were pre-incubated with seven different concentrations of SHY-01 (SHY, 0.03–2.0 mM) or metformin hydrochloride (Met) for 1 h, followed by LPS treatment for another 24 h. Supernatants were collected for nitrite analysis. Nonlinear regression was fit to the inhibition of SHY (blue line) and Met (red line). **(B)** Percentage of NO inhibition, **(C)** TNF-α protein level and **(D)** IL-1β protein level in the supernatant of BV-2 microglia cells pre-incubated with AMPK inhibitor (Compound C, CC, 10 μM) for 1 h followed by SHY-01 (SHY, 1 mM) and LPS (200 ng/ml) administration. Data were expressed as mean ± SD (*n* = 3) and analyzed using one-way ANOVA followed by a Dunnett’s post hoc test.^ ∗∗^ *p* < 0.01 versus the LPS group, ^##^ *p* < 0.01 versus the Met group.

Inflammatory mediators released by microglia induce secondary injury to the ischemic brain. We found that SHY-01 administration followed by LPS treatment effectively attenuated nitrite release with IC_50_ 302 μM, while IC_50_ for metformin hydrochloride was 1,070 μM (Figure 2A). Pre-incubation with compound C, a selective inhibitor of AMPK, abolished the inhibitory effect of SHY-01 on the LPS-induced expression of pro-inflammatory factors, such as nitrite, TNF-α, and IL-1β (Figures 2B–D) in BV-2 cells. These results demonstrated that the anti-inflammatory effect of SHY-01 is dependent on AMPK activation in BV-2 microglial cells.

### Pharmacokinetics of SHY-01 *In Vitro* and *In Vivo*

**FIGURE 3:**
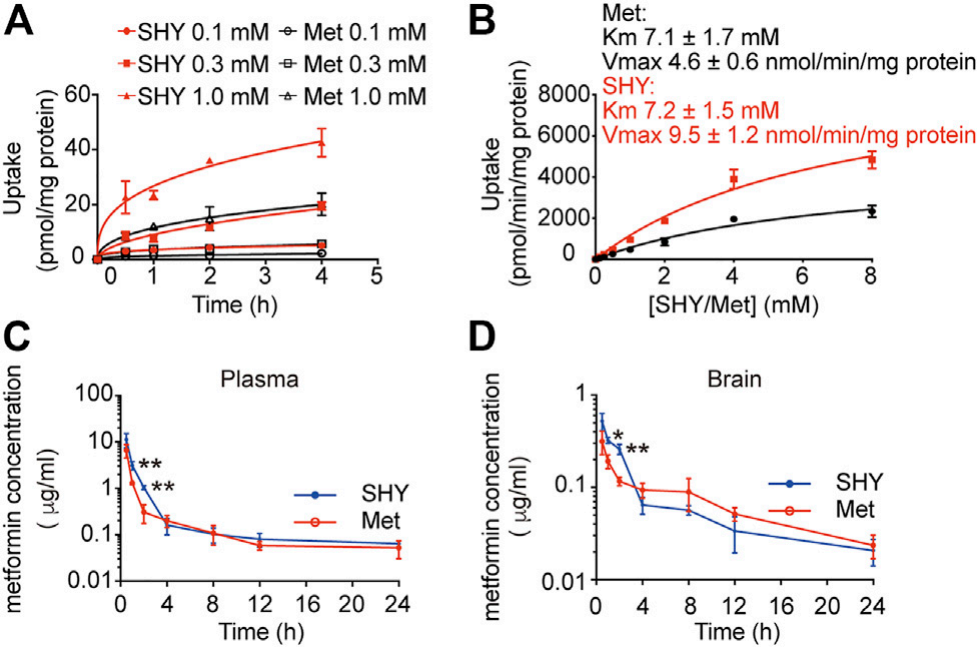
Pharmacokinetic profiles of metformin in microglial cells *in vitro* and rats *in vivo*. **(A)** BV-2 microglial cells were incubated with different concentration of SHY-01 (SHY) or metformin hydrochloride (Met) for the indicated periods, supernatants were discarded and cells were lysed for intracellular metformin analysis by LC-MS. Data were expressed as mean ± SD (*n* = 3). **(B)** Uptake of metformin (15 min) into BV-2 microglia cells incubated with different concentrations of SHY-01 (SHY) or metformin hydrochloride (Met). The Michaelis-Menten equation with one saturable component was fit to the corrected uptake rate and the estimated Km and Vmax values are presented. **(C)** Plasma and brain concentration-time profile of metformin after intraperitoneal injection of SHY-01 in rats. SHY-01 (SHY, 50 mg/kg) or metformin hydrochloride (Met, 50 mg/kg) were administrated (*i.p.*) to rats. Blood samples were taken from fundus venous plexus at 0, 15, 30, and 60 min, 2, 4, 8, 12, and 24 h after injections. Concentrations of metformin in plasma was analyzed by LC-MS/MS. **(D)** Rats were sacrificed and brains were assessed at 0, 15, 30, and 60 min, 2, 4, 8, 12, and 24 h after injections. Concentrations of metformin in brain tissue were analyzed by LC-MS/MS. Results are expressed as mean ± SD (*n* = 3 rats/group). Two-tailed unpaired Students’ *t*-test was used to analyzing the difference between SHY- and Met-treated groups in each time point. ^∗^ *p* < 0.05, ^∗∗^ *p* < 0.01 versus the Met-treated group.

To elucidate the differential effects of SHY-01 in relation to metformin hydrochloride (Metformin), we investigated the cellular pharmacokinetics in BV-2 microglia. Cells were incubated with different concentrations of SHY-01 or metformin, respectively. At designated times, LC-MS/MS analysis was conducted to determine metformin concentration. SHY-01 treatment resulted in elevated intracellular metformin contents as compared to that of metformin hydrochloride (Figure 3A). Moreover, the accumulation of metformin following SHY-01 treatment was more rapid than treatment with the same concentration of metformin hydrochloride (Figure 3B). We also measured the Vmax of metformin, which was 9.5 ± 1.2 nmol/min/mg protein for SHY-01 and 4.6 ± 0.6 nmol/min/mg protein for metformin hydrochloride. The rapid accumulation of metformin and increased Vmax following SHY-01 treatment were in agreement with the observed more rapid and more potent activation of AMPK as shown in Figure 1. To explore the pharmacokinetic profiles of SHY-01 and metformin hydrochloride *in vivo*, rats were injected intraperitoneally with either SHY-01 (50 mg/kg) or metformin hydrochloride (50 mg/kg)and blood and brain samples were collected at selected times, as described in the Methods. The plasma concentration-time profile of metformin is shown in Figure 3C. The maximum plasma concentration (C_max_), half-life (*t*_1/2_), and the area under the curve (AUC)_0∼∞_ of SHY-01 and metformin hydrochloride were 11.29 ± 3.9 vs. 6.63 ± 2.10 μg/L, 8.22 ± 5.52 vs. 7.72 ± 1.48 h, and 122.3 ± 0.39 vs. 71.66 ± 2.75 h·μg/L, respectively. Additional pharmacokinetic parameters are listed in Table 1. The brain distribution of metformin is summarized in Table 2. Metformin concentrations in the brain of SHY-01-treated rats were significantly higher than those in the metformin hydrochloride-treated rats at 1 h (0.32 ± 0.023 vs. 0.19 ± 0.032 μg/g; *p* = 0.005) and 2 h (0.25 ± 0.032 vs. 0.11 ± 0.012 μg/g; *p* = 0.002) after drug administration (Figure 3D). These results indicated that SHY-01 is more effective and rapidly enters the brain than metformin hydrochloride.

**TABLE 1:** The pharmacokinetic profiles of SHY-01 in rats with *i.p.* administration of 50 mg/kg metformin threonate (SHY-01) or metformin hydrochloride.

Parameters	Unit	SHY-01	Metformin hydrochloride
AUC (0−t)	hr·μg/ml	11.47 ± 0.035	6.59 ± 0.27
AUC (0−∞)	hr·μg/ml	12.24 ± 0.040	7.17 ± 0.28
MRT (0−t)	H	2.69 ± 0.45	3.48 ± 0.65
MRT (0−∞)	H	5.06 ± 2.54	6.06 ± 1.73
*t*_1/2_	H	8.22 ± 5.524	7.72 ± 1.48
*t*_max_	H	0.5	0.5
CLz	ml/hr/kg	4,116.73 ± 422.20	7,068.89 ± 949.45
Vz	ml/kg	50,055.01 ± 36,787.23	79,683.93 ± 23,176.87
C_max_	μg/ml	11.30 ± 3.90	6.64 ± 2.11

*AUC (0−t), Area under concentration-time curve from time zero to the last measured concentration; AUC (0−∞), Area under concentration-time curve from time zero to infinite; MRT (0−t), Mean retention time from zero to the last measures concentration; MRT (0−∞), Mean retention time from zero to infinite; T1/2: Half lifetime; Tmax, Time to maximum plasma concentration; CLz, Clearence; Vz, Apparent volume of distribution; Cmax, Maximum plasma concentration.

Forty-two SD male rats were intraperitoneally administrated with SHY-01 (50 mg/kg) or Metformin hydrochloride (50 mg/kg). Blood samples were collected from orbits at 0.5, 1, 2, 4, 8, 12, and 24 h. The concentration of metformin in plasma were analyzed by LC-MS/MS. The pharmacokinetic parameters of SHY-01 and metformin hydrochloride were calculated accordingly. The results are presented as means ± SD (*n* = 3/group).

**TABLE 2:** Rat brain distribution of metformin treated with metformin threonate (SHY-01) or metformin hydrochloride (50 mg/kg, *i.p.*).

Parameters	Unit	SHY-01	Metformin hydrochloride
AUC (0−t)	hr·μg/ml	1.87 ± 0.23	1.60 ± 0.18
AUC (0−∞)	hr·μg/ml	2.05 ± 0.18	1.89 ± 0.08
MRT (0−t)	h	5.73 ± 0.63	7.71 ± 0.45
MRT (0−∞)	h	8.19 ± 0.66	12.20 ± 2.90
*t*_1/2_	h	8.26 ± 1.88	6.03 ± 0.28
*t*_max_	h	0.5	0.5
CLz	ml/hr/kg	24.48 ± 2.25	26.46 ± 1.10
Vz	ml/kg	213.80 ± 29.93	31.65 ± 78.86
C_max_	μg/ml	0.52 ± 0.11	0.32 ± 0.08

Forty-two SD male rats were intraperitoneally administrated with SHY-01 (50 mg/kg) or Metformin hydrochloride (50 mg/kg). Brain samples were collected after sacrifice at 0.5, 1, 2, 4, 8, 12, and 24 h (three rats each time point per drug). The concentration of metformin in brain were analyzed by LC-MS/MS. The pharmacokinetic parameters of SHY-01 and metformin hydrochloride were calculated accordingly. The results are presented as means ± SD (*n* = 3/group).

### SHY-01 Treatment Reduces Infarct Volume and Improves Neurological Deficits on tMCAO-Induced Rats

**FIGURE 4:**
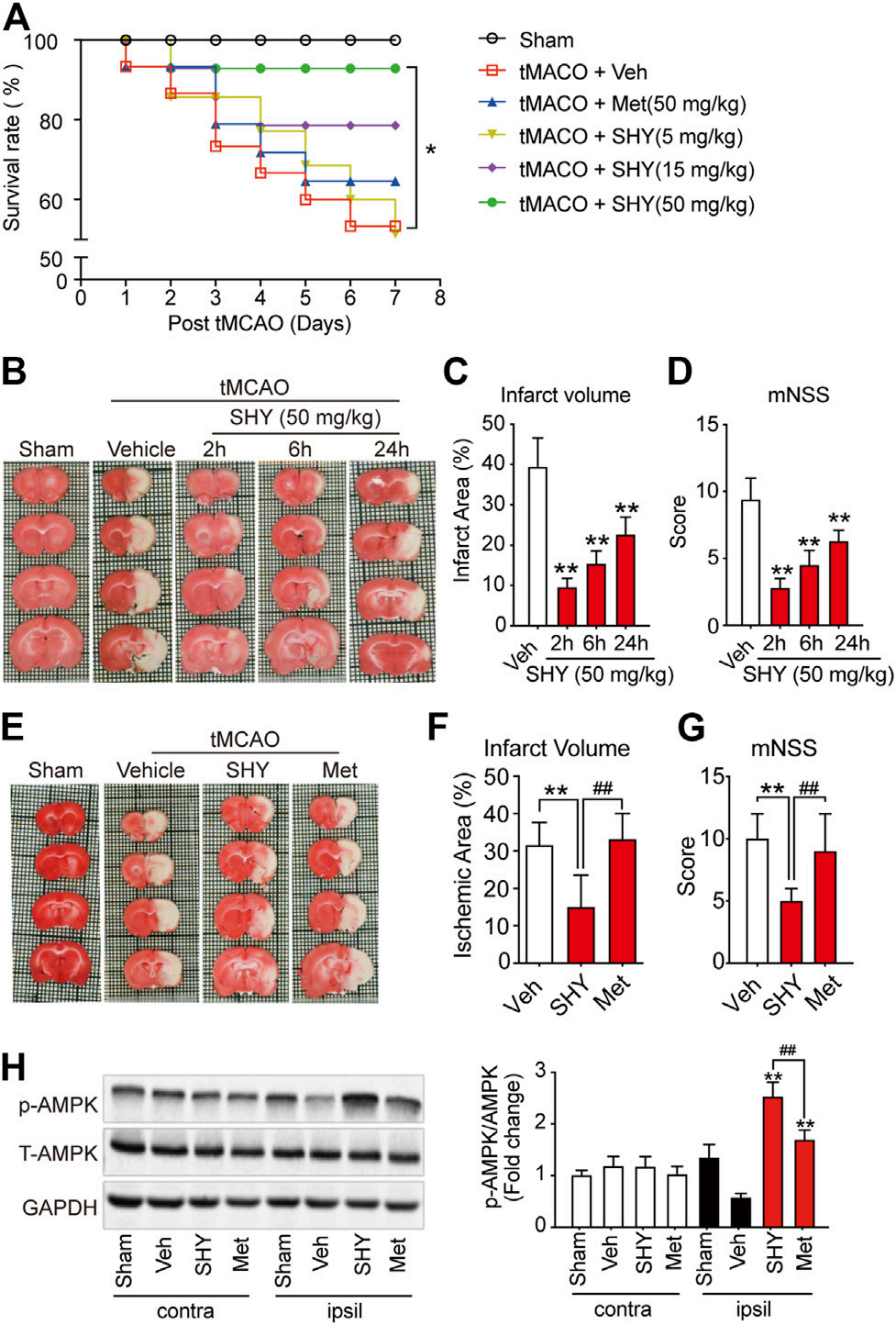
SHY-01 ameliorates infarct area in tMCAO-induced cerebral ischemic stroke. **(A)** SHY-01 (SHY, 5, 15, or 50 mg/kg) or metformin hydrochloride (Met, 50 mg/kg) were administrated (*i.p.*) every 24 h after tMCAO, saline was used as vehicle control (Veh), survival rate of rats was recorded 7 days post tMCAO. *n* = 15 rats/group, Kaplan-Meier analysis followed by a Cox regression test to compare differences between each group. **(B)** SHY-01 (50 mg/kg) was administrated (*i.p*.) at 2, 6, or 24 h after reperfusion. The rats were euthanized at 72 h after tMCAO and representative images of brain sections stained with TTC are illustrated. **(C)** The percentage of the infarct area in whole brain were statistically analyzed, **(D)** mNSS score in each group were evaluated for each group. Results are expressed as means ± SD. *n* = 7–8 rats/group for infarct area and mNSS score determination. Statistical analysis was performed using one-way ANOVA with Dunnett’s post hoc tests. ∗*p* < 0.05, ∗∗*p* < 0.01, versus the vehicle group. **(E)** SHY-01 (SHY, 50 mg/kg) or metformin hydrochloride (Met, 50 mg/kg) was administrated (*i.p.*) at every 24 h after reperfusion, rats were sacrificed at 72 h after tMCAO. Representative images of brain sections stained with TTC were illustrated and **(F)** percentage of the infarct area in whole brain were statistically analyzed, **(G)** mNSS score in each group were evaluated as described in material and methods. *n* = 7–8 rats/group for infarct area and mNSS score determination. Statistical analysis was performed using one-way ANOVA with Dunnett’s post hoc tests. ^∗^ *p* < 0.05, ^∗∗^ *p* < 0.01, versus the vehicle group. ^##^ *p* < 0.01, versus metformin hydrochloride group. **(H)** Immunoblotting of AMPK phosphorylation (Thr 172) level in the contralateral (contra) or ipsilateral (ipsil) of each group and relative phospho-AMPK levels were analyzed using one-way ANOVA with Dunnett’s post hoc tests. ^∗^ *p* < 0.05, ^∗∗^ *p* < 0.01, versus the vehicle group. ^##^ *p* < 0.01, versus metformin hydrochloride group.

We have hypothesized that the lack of protective effect of metformin on acute cerebral ischemia may be attributed to a slow accumulation of metformin in the brain cells. Considering that SHY-01 accumulates more rapidly in cells *in vitro* and in rat brains, and this leads to a more potent and faster activation of AMPK than with metformin hydrochloride (Figures 1–3), we thus thought to test if SHY-01 will produce beneficial effects on acute cerebral ischemia. Rats were subjected to tMCAO followed by 5, 15, or 50 mg/kg of SHY-01 or 50 mg/kg of metformin hydrochloride administration daily for 7 days. We found that the SHY-01 treatment dose-dependently improved the survival rate of ischemic rats in which an improvement was already observed with 15 mg/kg SHY-01 treatment, while 50 mg/kg SHY-01 present significant elevation as compared to that of metformin hydrochloride (Figure 4A). In association with this observation, we also found a dose-dependent decrease in infarct volume and improvement in neurologic deficits (Supplementary Figure S1). In addition, when we tested the time window of SHY-01 treatment, we found similar protective effects of SHY-01 on acute ischemic stroke at 2, 6, and 24 h after ischemic stroke, as indicated by the reduced infarct area (Figures 4B,C) and improved neurologic score (Figure 4D). These data suggested that SHY-01 has a beneficial effect on tMCAO-induced acute ischemic stroke rats. To further validate the protective effect of SHY-01, we compared infarct area and neurologic deficits of ischemic stroke rats treated with SHY-01 or metformin hydrochloride at 72 h after reperfusion. Rats were administrated with SHY-01 (SHY, 50 mg/kg, *i.p.*) or metformin hydrochloride (Met, 50 mg/kg, *i.p.*) every 24 h post-reperfusion on tMCAO-induced rats. As shown in Figure 4, the application of metformin hydrochloride did not produce any beneficial effects on infarct volume or neurologic deficits, whereas the same dose of SHY-01 significantly improved both outcomes (Figures 4E,F). In association with the results, the neurological score reflecting rat’s behavior including sensory, motor, balance, and reflex ability also showed that SHY-01 but not metformin hydrochloride, markedly preserved neurologic functions in rats subjected to acute cerebral ischemia (Figure 4G). Furthermore, we found that both SHY-01 and metformin hydrochloride treatment could effectively enhance AMPK phosphorylation, but the SHY-01 group exhibited significantly higher p-AMPK expression than metformin hydrochloride group (Figure 4H). All these data clearly indicated that SHY-01 administration produced protective effects on acute ischemic stroke.

### SHY-01 Administration Elicits Potent Neuroprotection Either *via* Suppression of Microglia Activation or Direct Neuroprotection

**FIGURE 5:**
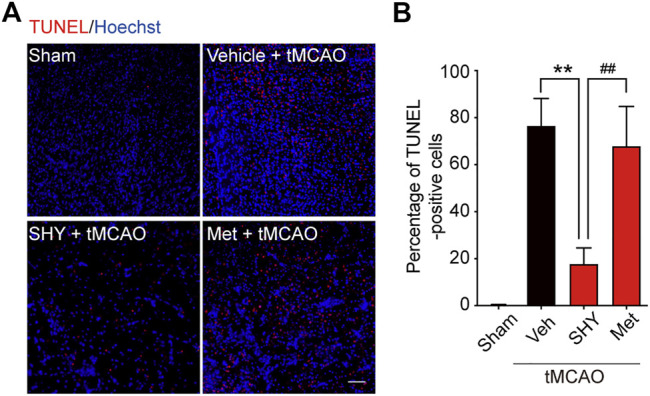
SHY-01 administration displays neuronal protection post-ischemia. SHY-01 (SHY, 50 mg/kg) or metformin hydrochloride (Met, 50 mg/kg) was administrated (*i.p.*) every 24 h post-reperfusion, saline was used as vehicle control, rats were sacrificed at 72 h after tMCAO, and brains were assessed for immunofluorescence analysis with frozen sections. **(A)** Apoptotic cells in the ischemic striatum were stained with TUNEL (red) as detailed in the Methods, nuclei were stained with Hoechst (blue), scale bar 50 μm. **(B)** Percentage of TUNEL-positive apoptotic cells among total cells were determined with ImageJ software and statistical analysis were presented. Data are expressed as mean ± SD, *n* = 5, statistical analysis was performed with one-way ANOVA followed by a Dunnett’s post hoc tests. **p < 0.01 versus the tMCAO with vehicle treatment group, ^##^ *p* < 0.01 tMCAO treated with Met versus SHY-01-treated group.

We first evaluated the neuroprotective effects of SHY-01 on tMCAO by visualizing the apoptotic cells using TUNEL staining. As shown in Figure 5, the number of TUNEL-positive cells at the ischemic site in tMCAO rats with SHY-01 treatment group was significantly lower than in either the vehicle- or metformin hydrochloride-treated group (Figure 5A). Quantitative analysis for the percentage of TUNEL-positive cells among total cells in the observed area indicated that the apoptotic index in SHY-01 treatment group was markedly decreased as compared to the vehicle- or metformin hydrochloride-treated group (Figure 5B). This data clearly revealed the superior neuroprotective effects of SHY-01.

**FIGURE 6:**
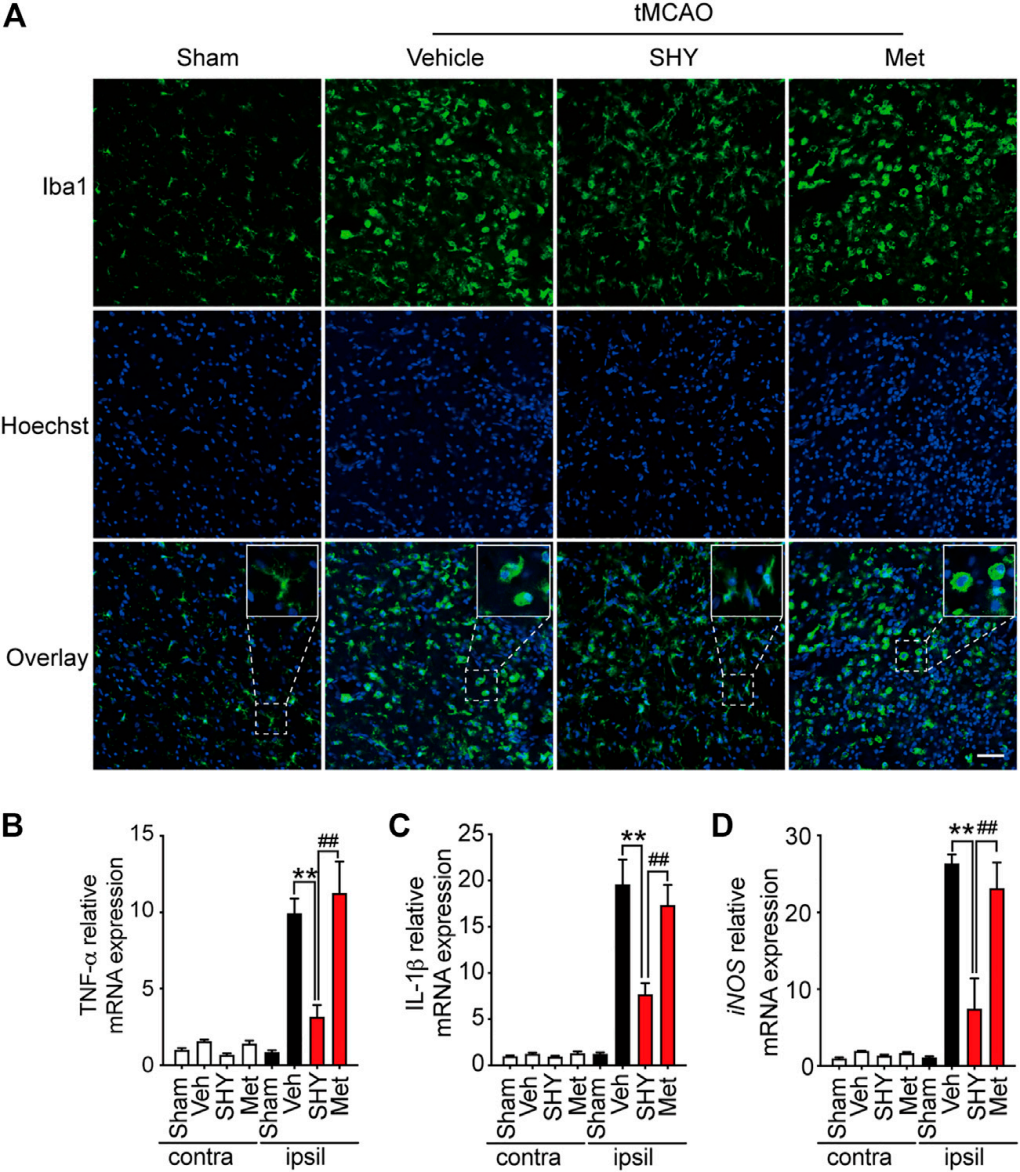
SHY-01 administration suppresses microglia activation in the ischemic striatum. SHY-01 (SHY, 50 mg/kg) or metformin hydrochloride (Met, 50 mg/kg) was administrated (*i.p.*) daily post reperfusion, saline was used as vehicle control, rats were sacrificed at 72 h after tMCAO. **(A)** Brains were assessed for immunofluorescence analysis with frozen sections. Microglia were marked with Iba-1 (green) and nuclei were stained with Hoechst (blue). Representative images detected with confocal microscopy show cellular morphology in the ischemic striatum, *n* = 4 rats per group, scale bar: 20 μm. Brains were lysed with Trizol to extract total message RNA, then were reverse transcribed to determine mRNA level of **(B)** TNF-α, **(C)** IL-1β, and **(D)** iNOS. GAPDH was used as interior reference. Data were expressed as mean ± SD, *n* = 5 per group, statistical analysis using one-way ANOVA followed by Dunnett’s post hoc tests. ^∗∗^ *p* < 0.01 versus the tMCAO with vehicle treatment group, ^##^ *p* < 0.01 versus the tMCAO with SHY treatment group.

**FIGURE 7:**
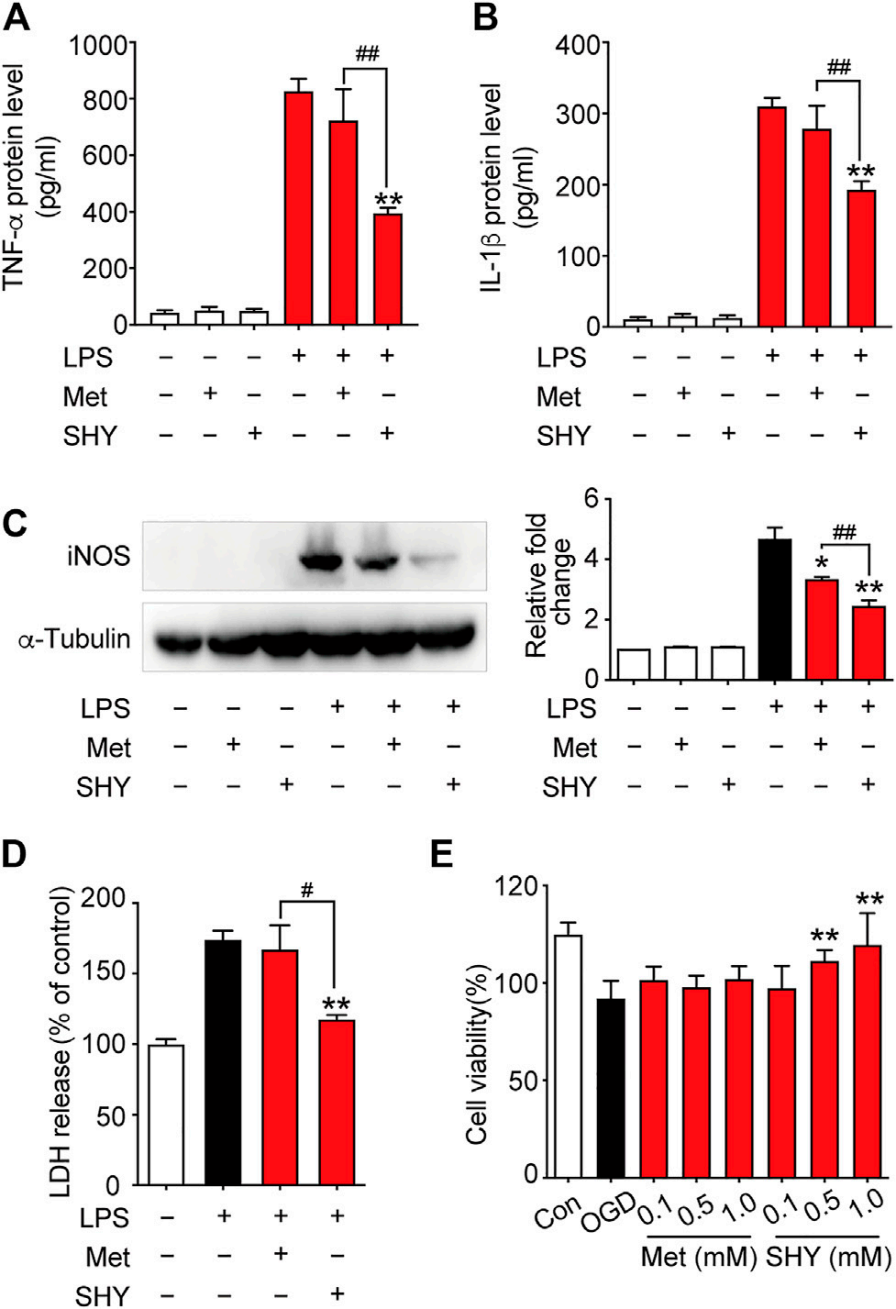
SHY-01 protects against neuronal damage *via* suppression of microglia activation or direct neuroprotection. BV-2 microglial cells were pre-incubated with SHY-01 (SHY, 1 mM) or metformin hydrochloride (Met, 1 mM) for 1 h followed with LPS (200 ng/ml) treatment. Supernatants of microglia were collected after 24 h for **(A)** TNF-α and **(B)** IL-1β protein level measurement with ELISA. Data were presented as mean ± SD, statistical analysis was performed with one-way ANOVA followed by a Dunnett’s post hoc tests, ^∗∗^ *p* < 0.01 versus LPS group; ^##^ *p* < 0.01, versus LPS with SHY-treated group. **(C)** BV-2 microglial cells were collected after 12 h of LPS treatment for iNOS protein level measurement with Immunoblotting. *n* = 3, data are presented as means ± SD, statistical analysis was performed with one-way ANOVA, followed by a Dunnett’s post hoc tests, ^∗^ *p* < 0.05, ^∗∗^ *p* < 0.01 versus LPS group; ^##^ *p* < 0.01, versus LPS with SHY-treated group. **(D)** Rat primary microglia were treated with SHY (1 mM) or Met (1 mM) for 1 h, followed with LPS (200 ng/ml) incubation for 24 h. Conditioned medium was then collected to stimulate rat primary cortical neurons for another 24 h, supernatant was harvested for LDH analysis. Results are expressed as mean ± SD (*n* = 5). Data are presented as mean ± SD, statistical analysis was performed with one-way ANOVA, followed by a Dunnett’s post hoc tests, ^∗∗^ *p* < 0.01 versus LPS group; ^#^ *p* < 0.05, versus LPS with SHY-treated group. **(E)** Primary cortical neurons were pre-treated with SHY or Met at 0.1, 0.5, and 1.0 mM for 24 h. The cultures were then subjected to oxygen and glucose deprivation (OGD) for 3 h followed by reperfusion with normal culture medium for another 24 h. Cell viability was analyzed by MTT assay, results are expressed as mean ± SD (*n* = 8). Statistical analysis was performed with one-way ANOVA, followed by a Dunnett’s post hoc tests. ^∗∗^ *p* < 0.01 versus OGD group.

We then further elucidated the mechanism underlying the superior protection of SHY-01. It is well known that microglia activation plays a critical role in ischemia-induced neuronal damage, which contributes to the pathologic development of stroke. Accordingly, we evaluated the effect of SHY-01 on microglial activation in tMCAO rats by immunohistochemistry. We found that SHY-01 treatment exerted stronger inhibition on microglial activation, as indicated either by cell morphological features or Iba-1 positive cell numbers. Microglial cells in the ischemic striatum presented as amoeboid cell shape in the metformin hydrochloride-treated ischemic rats, whereas in the ischemic rats with SHY-01 administration, microglial cells transformed into ramified shape with small soma, very fine and long processes (Figure 6A). Moreover, we found that the expression of pro-inflammatory modulators, such as TNF-α (Figure 6B), IL-1β (Figure 6C), and iNOS (Figure 6D) in the ischemic striatum of rats subjected to tMCAO was dramatically suppressed by SHY-01 treatment, which elicited more potent inhibition on neuroinflammation as compared to that of metformin hydrochloride. In addition, SHY-01 elicited a stronger effect on the inhibition of LPS-induced pro-inflammatory cytokine expression (Figures 7A–C) for TNF-α, IL-1β, and iNOS, respectively than metformin hydrochloride. To further test how the potent inhibitory effect of SHY-01 on neuroinflammation contributes to neuroprotection, we employed a conditioned neuronal culture system to detect if the conditional medium from SHY-01 treated primary microglia cells activated by LPS could protect primary cultured neurons. As shown in Figure 7D, the application of SHY-01 treated conditioned medium produced a more potent protective effect as evidenced by a remarkably decrease in LDH release in response to LPS as compared to either control or metformin hydrochloride treatment. This indicated that SHY-01 inhibited microglial activation indeed contributed to the potent neuroprotection.

We next wondered if SHY-01 also elicits a direct neuroprotection, we examined the potential protective effect of SHY-01 on rat primary cortical neurons subjected to oxygen and glucose deprivation (OGD), which is a widely used *in vitro* cellular model of ischemic condition *in vivo*. The result showed that SHY-01 significantly prevented OGD-induced neuronal injury in cultured primary cortical neurons at 0.5 and 1 mM (Figure 7E), whereas the same dose of metformin hydrochloride did not elicit such an effect. These results, therefore, clearly revealed that SHY-01 produced powerful and superior neuroprotection either by inhibition of microglia activation or direct protection. However, the latter needs further investigation.

### Chronic SHY-01 Treatment Improves Long-Term Functional Recovery of tMCAO Rats

**FIGURE 8:**
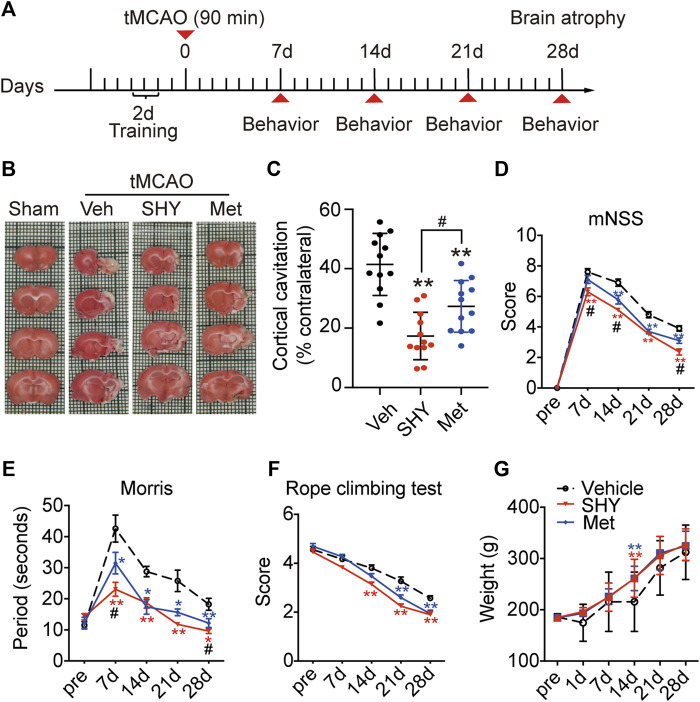
SHY-01 promotes long-term functional recovery of rats with tMCAO. SHY-01 (SHY, 50 mg/kg/day) or metformin hydrochloride (Met, 50 mg/kg/day) was administrated (*i.p.*) every 24 h post reperfusion, saline was used as vehicle control, rats were sacrificed at 4 weeks after tMCAO. **(A)** Timeline of SHY or Met treatments during neurologic recovery over the period of 4 weeks post-stroke. **(B)** Representative images of rat brain sections stained with TTC. **(C)** Brain atrophy was quantified, and **(D)** neurologic scores were analyzed in each group. *n* = 12 rats/group, data are expressed as mean ± SD, statistical analysis was performed with one-way ANOVA, followed by a Dunnett’s post hoc tests. ^∗∗^ *p* < 0.01 versus tMCAO treated with vehicle group; ^#^ *p* < 0.05, versus tMCAO with SHY-treated group. Behaviors of **(E)** Morris water maze test and **(F)** rope climbing test were evaluated, **(G)** weight changes were recorded for 4 weeks post-ischemia. Results are expressed as medium ± SEM (*n* = 8/group). Statistical analysis in each time point was performed by statistical analysis was performed with one-way ANOVA, followed by a Dunnett’s post hoc tests. ^∗^ *p* < 0.05, ^∗∗^ *p* < 0.01 versus vehicle group; ^#^ *p* < 0.05 versus SHY-treated group.

Our results described so far, demonstrated that, unlike metformin hydrochloride, SHY-01 elicits a protective effect on the acute phase of experimental ischemic stroke. We next investigated the effect of chronic SHY-01 administration on the long-term functional recovery in experimental ischemic stroke. The paradigm used in these experiments is depicted in Figure 8A. The result demonstrated that daily administration of SHY-01 or metformin hydrochloride for four consecutive weeks effectively reduced the atrophic volume of the ischemic brain (Figure 8B). Interestingly, SHY-01 treatment produced a more potent reduction in brain atrophy (Figure 8C). Consistent with the results, SHY-01 also produced significantly better improvement in the neurologic score (Figure 8D) and in spatial learning in the Morris water maze test (Figure 8E) as compared to that of the metformin hydrochloride treatment, whereas Rope climbing test (Figure 8F) and body weight changes (Figure 8G) did not differ between the two treatments. Taken all together, these data clearly indicated that, compared to metformin hydrochloride, SHY-01 exhibited significantly better beneficial effects than metformin hydrochloride on the long-term functional recovery in tMCAO rats. These data again revealed the superior efficacy of SHY-01 in ischemic stroke treatment.

## Discussion

Cerebral ischemia is the most prevalent form of stroke and treatment for the disease remains a huge challenge. It is generally believed that neuroinflammation plays a key role in the pathological development of stroke. Therefore, modulation of neuroinflammation is considered as a critical approach for ischemic stroke treatment. On the one hand, post-stroke activation of neuroinflammation is a defensive response to ischemic insult but, on another hand, neuroinflammation also contributes to the secondary brain damage. In the current study, we took advantage of the recently developed patented derivative of metformin (SHY-01), which exhibits improved pharmacokinetic profiles with rapid accumulation in cells, stronger activation of AMPK, and tested the beneficial effects on ischemic stroke. We found that, unlike classical metformin hydrochloride, SHY-01 exhibited strong protection during the acute phase of ischemic stroke as evidenced by reduced infarct volume and improved neurologic scores. We found that SHY-01 exerted rapid and strong inhibition on neuroinflammation as compared to metformin hydrochloride. The underlying mechanism may be associated with the stronger inhibition effect of SHY-01 on M1 microglia and the promotion of M2 phenotypes that is attributed to its improved pharmacokinetic profiles. We further demonstrated that chronic application of SHY-01 produced a better beneficial effect in long-term functional recovery than that of metformin hydrochloride in ischemic rats. The present study provided clear evidence that metformin threonate elicited superior therapeutic effects on cerebral ischemic stroke.

We and others have reported that chronic post-stroke administration of metformin significantly promotes long-term functional recovery of ischemic stroke mice (Jin et al., 2014; Zhu et al., 2015; Zemgulyte et al., 2021) that reproduced the beneficial effects of metformin observed in diabetes in which metformin taking patients who typically have a lower incidence of stroke or less severe neurologic functional deficits if they experience a stroke (Westphal et al., 2020). Moreover, the beneficial effects of metformin were also reported in non-diabetic stroke patients (Jadhav et al., 2006). However, it is noted that, in spite of the beneficial effects of chronic metformin on long-term neurologic recovery, administration of the drug produced no significant benefit in acute stroke either in the clinical application or in experimental studies (Jin et al., 2014; Mima et al., 2016), which limited the application of metformin in stroke. It is known that it usually requires a few hours for metformin hydrochloride to significantly activates AMPK (Ma et al., 2014). We, therefore, hypothesized that the lack of beneficial effect of metformin hydrochloride in the acute phase of ischemic stroke could be due to the slow intracellular accumulation of metformin. We thus synthesized different radical acid of metformin and found that SHY-01, metformin L-threonate rapidly accumulates in cells (Figures 3A,B). This, in turn, leads to rapid activation of AMPK within 30 min after SHY-01 treatment, which is significantly faster as compared to that of metformin hydrochloride, which usually requires 4 h or longer treatment (Ma et al., 2014). In addition to pharmacokinetic differences, we found that, in comparison with classic metformin hydrochloride, SHY-01 also exhibited significant advantages in terms of pharmacological effects: 1) SHY-01 elicited stronger inhibition on neuroinflammation and produced stronger neuroprotection *in vitro* and *in vivo*; 2) post-stroke administration of SHY-01 produced protective effect during the acute phase of tMCAO as evidenced by decreased infarct volume and mortality, whereas the same dose of metformin hydrochloride treatment failed to produce the protection; 3) chronic SHY-01 administration had superior effects on long-term neurologic functional recovery in tMCAO rats in relation to metformin hydrochloride; 4) lastly, we showed that the protection on the ischemic stroke of SHY-01 is mediated by AMPK. The improved pharmacokinetic profiles render the drug to elicit rapidly and more potent activation on AMPK. Collectively, these results clearly demonstrated that SHY-01 exhibited superior beneficial pharmacological effects on an experimental ischemic stroke when compared to metformin hydrochloride. Our data, therefore, revealed that SHY-01 may be a promising drug candidate for stroke treatment.

Microglia/macrophages play a critical role in the pathology and prognosis of ischemic stroke (Xiong et al., 2016; Kluge et al., 2017). We previously have shown that metformin hydrochloride exerts neuroprotection on ischemic stroke mainly via inhibition of microglial activation and promotion of the polarization of M2 phenotypes (Jin et al., 2014; Jia et al., 2015). We showed that here, in association with its rapid and stronger activation of AMPK, SHY-01 strongly inhibits microglia-mediated inflammation in both BV2 cells *in vitro* and in the ischemic brain (Figures 6, 7). AMPK is well known to be involved in the regulation of neuroinflammation by acting as a metabolic sensor to induce metabolic reprograming (Frasch, 2014; Liu et al., 2014; Zhu et al., 2015; Kodali et al., 2021). AMPK is reported to inhibit M1 phenotypes and promote M2 polarization, thus inhibiting neuroinflammation (Ji et al., 2018). We confirmed this finding in BV2 cells and in tMCAO animals. Moreover, SHY-01 inhibited the LPS-induced RNS production is significantly more potent (IC_50_: 302 μM) than that of metformin hydrochloride (IC_50_: 1,070 μM) in BV2 cells (Figure 2A). In agreement with this observation, SHY-01-treated conditioned medium from LPS-activated BV2 cells induced significantly less cell death, thereby providing more effective neuroprotection than metformin hydrochloride (Figure 7D). In addition to demonstrating a superior inhibitory effect of SHY-01 on neuroinflammation, we provided preliminary evidence that SHY-01 exhibits a direct neuroprotection in OGD cortical neuronal cell cultures (Figure 7E). It was previously shown that metformin hydrochloride modulates neuronal apoptosis (Chen X. et al., 2020; Zhang et al., 2020) and involves in the cell survival modulations (Yan et al., 2017; Kodali et al., 2021). We previously also showed that metformin hydrochloride treatment altered autophagic activity in dopaminergic neurons (Yan et al., 2017). However, the detailed direct pharmacological effects of SHY-01 on neuronal protection remain to be further studied. It should be noted that numerous compounds have been demonstrated to be neuroprotectants in preclinical models, but rare were able to translate into the clinical application (O’Collins et al., 2011). The reason behind this is not known but some factors, such as initial disease severity, short treatment window, and complex subtypes may attribute to the various outcomes. Therefore, establishing an objective measurement of the robustness of treatment effect, such as pPREDICTS might be a solution to find an effective agent in clinical trials (Kent and Mandava, 2016), and future trials of the putative neuroprotectants combined with reperfusion therapies might be conducted in stroke patients (Chamorro et al., 2021). It is also pointed out, as many studies indicated (Jin et al., 2014; Zhu et al., 2015; Zemgulyte et al., 2021), that we recorded a dramatic reduction in infarct volume but improvement in long-term neurological recovery, however, was less promising.

## Conclusion

The present data reveal that SHY-01 (metformin threonate) produces a superior pharmacological efficacy in the treatment of ischemic stroke, which is supported by its improved pharmacokinetic profiles. As such, SHY-01 may be a promising candidate drug for stroke treatment. However, the underlined mechanism for how threonate improves the metformin uptake to the crossing plasma membrane is currently unknown.

## Data Availability

The original contributions presented in the study are included in the article/Supplementary Material; further inquiries can be directed to the corresponding author.

## Abbreviations

AMPK, AMP‐activated protein kinase; LPS, lipopolysaccharide; mNSS, modified neurological severity scores; SHY-01, metformin threonate; tMCAO, transient middle cerebral artery occlusion.
