# Supplementary figures and images for: Development and Evaluation of Novel Metformin Derivative Metformin Threonate for Brain Ischemia Treatment
Source: Front Pharmacol. 2022 Jun 21;13:879690. doi: 10.3389/fphar.2022.879690 (PMC9253272; doi:10.3389/fphar.2022.879690)

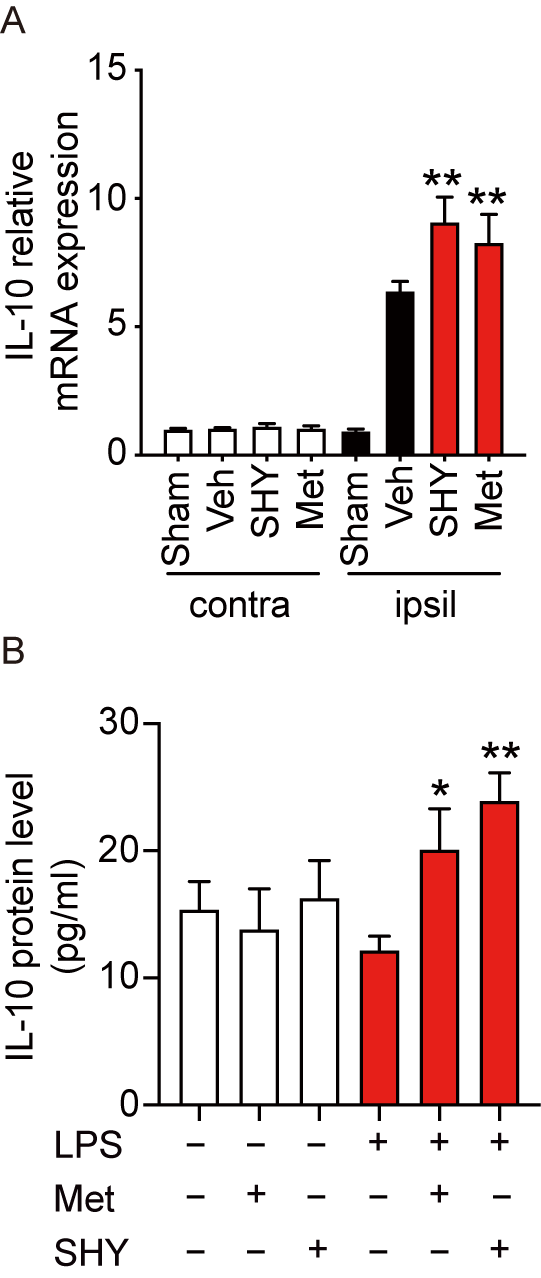

Supplement: Supplementary file 1 [file Image2.TIF]

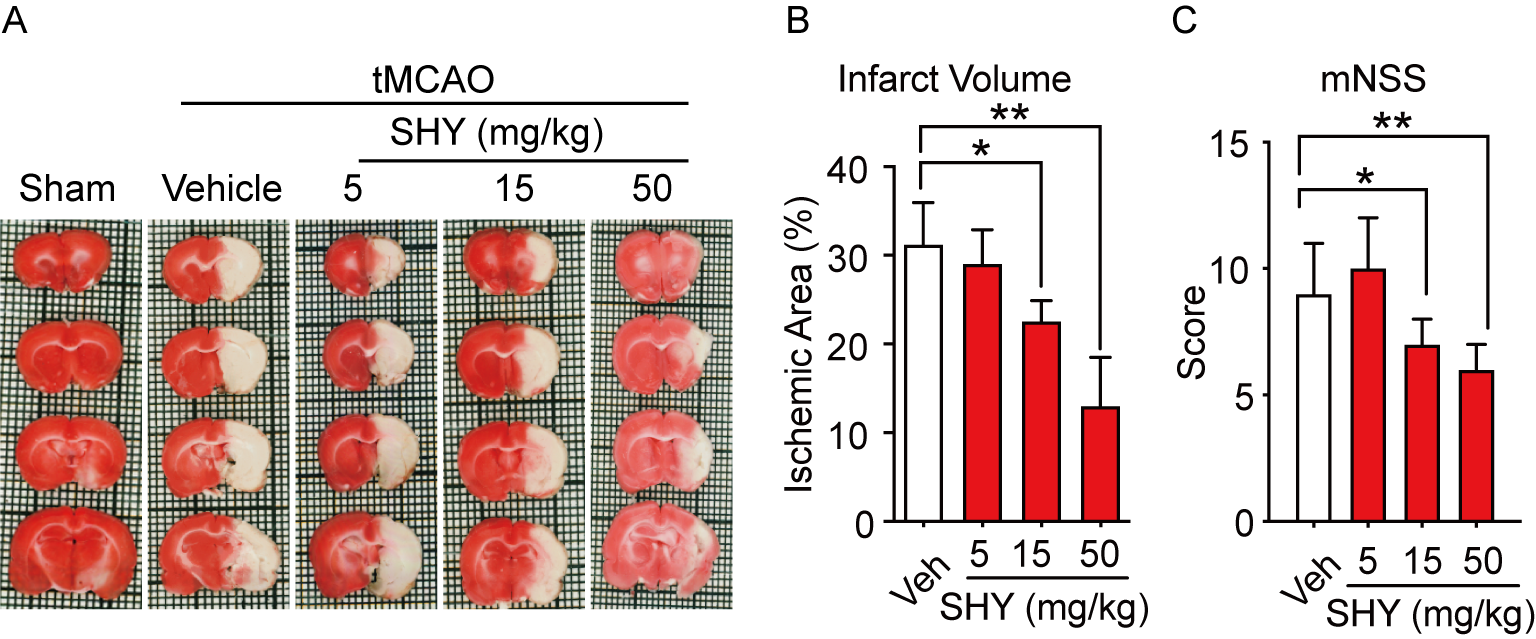

Supplement: Supplementary file 2 [file Image1.TIF]
