# Supplementary material for: Development and Evaluation of Novel Metformin Derivative Metformin Threonate for Brain Ischemia Treatment
Source: Front Pharmacol. 2022 Jun 21;13:879690. doi: 10.3389/fphar.2022.879690 (PMC9253272; doi:10.3389/fphar.2022.879690)
Supplement: Supplementary file 3 [file DataSheet1.docx]

**Supplemental Figure Legend**

**Supplemental Figure 1** SHY-01 produce protective effect dose-dependently on focal cerebral ischemia injury. SHY-01 (SHY, 5, 15, 50 mg/kg) were administrated (i.p.) every 24 hours after tMCAO, saline was used as vehicle control (Veh), rats were sacrificed 72 hours after tMCAO. (A) Representative images of brain sections staining with TTC were presented and (B) percentage of infarct area in whole brain were statistically analyzed. (C) mNSS score in each group were evaluated according to material and methods. Results are expressed as means±SD. Statistical analysis was performed using one-way ANOVAs with Dunnett’s post hoc tests, n = 8. **p*<0.05, ***p*<0.01 versus the vehicle group.

**Supplemental Figure 2** SHY-01 administration enhanced IL-10 expression in ischemic area of rats subjected to tMCAO. SHY-01 (SHY, 50mg/kg) or metformin hydrochloride (Met, 50mg/kg) was administrated (i.p.) every 24 h post reperfusion, saline was used as vehicle control, rats were sacrificed at 72 h after tMCAO. Brains were lysed with Trizol to extract total message RNA, then were reversed to analyzing mRNA level of IL-10. GAPDH was used as interior control. Data were expressed as mean ± SD, n = 5, statistical analysis using one-way ANOVA, followed by a Dunnett’s post hoc tests. *p<0.05, **p<0.01 versus the tMCAO with vehicle treatment group.
